# Metal Complexes as Promising Matrix Metalloproteinases Regulators

**DOI:** 10.3390/ijms24021258

**Published:** 2023-01-09

**Authors:** Yen Thi Nguyen, Namdoo Kim, Hyuck Jin Lee

**Affiliations:** 1 Department of Chemistry, Kongju National University, Gongju 32588, Chungcheongnam-do, Republic of Korea; 2 Department of Chemistry Education, Kongju National University, Gongju 32588, Chungcheongnam-do, Republic of Korea

**Keywords:** matrix metalloproteinases, metal complexes, cancer, Alzheimer’s disease

## Abstract

Nowadays, cancers and dementia, such as Alzheimer’s disease, are the most fatal causes of death. Many studies tried to understand the pathogenesis of those diseases clearly and develop a promising way to treat the diseases. Matrix metalloproteinases (MMPs) have been reported to be involved in the pathology of cancers and AD through tumor cell movement and amyloid degradation. Therefore, control of the levels and actions of MMPs, especially MMP-2 and MMP-9, is necessary to care for and/or cure cancer and AD. Various molecules have been examined for their potential application as regulators of MMPs expression and activity. Among the molecules, multiple metal complexes have shown advantages, including simple synthesis, less toxicity and specificity toward MMPs in cancer cells or in the brain. In this review, we summarize the recent studies and knowledge of metal complexes (e.g., Pt-, Ru-, Au-, Fe-, Cu-, Ni-, Zn-, and Sn-complexes) targeting MMPs and their potentials for treating and/or caring the most fatal human diseases, cancers and AD.

## 1. Introduction

### 1.1. Matrix Metalloproteinases in Cancers, and Alzheimer’s Disease

Matrix metalloproteinases (MMPs) are a family of Ca-dependent Zn-containing endopeptidases. The major function of MMPs is degrading extracellular matrix (EM) related to tissue repair/remodeling, development, and morphogenesis [1,2]. Usually, MMPs are in inactive forms by binding of Cys in the N-terminal pro-domain to Zn(II) in the active site [3]. When MMPs are in active forms, the enzymes are related to the pathology of various diseases (e.g., cancers, Alzheimer’s disease (AD), stroke, cardiovascular diseases, and rheumatoid) as presented in Table 1 [4,5,6,7,8].

Among MMPs, MMP-2 and MMP-9 have been reported to be involved in cancer progression and metastasis [9,10]. A large amount of active MMP-2 and MMP-9 was found in tumor tissues [11,12,13]. The activated MMP-2 and MMP-9 could promote tumor cell movement via degrading collagen type IV of the basement membrane [14]. Overexpression of MMP-2 could activate p38-MAPK/ML/SHP27 signaling, resulting in actin polymerization through cell migration [15]. Moreover, MMP-2 and MMP-9 could affect angiogenesis to enhance tumoral growth and development [16].

In addition, various studies revealed that MMP-2 and MMP-9 could affect amyloid-β (Aβ) aggregation and clearance [17,18,19,20,21,22]. MMP-2 and MMP-9 have been demonstrated to degrade soluble Aβ and amyloid plaques into less toxic fragments, possibly leading to the protection of neurons from amyloid toxicity [21,23]. Moreover, the inactivation of MMP-2 and MMP-9 could cause an increase in Aβ level [24]. Particularly, the overexpression of MMP-9 could promote the increase in soluble amyloid precursor protein a (sAPPα) along with a decrease in Aβ oligomers and the improvement of cognitive abilities of AD mice [25]. Moreover, it has been reported that the increase in MMP-9 activity could be related to proteolytic degradation of nerve growth factor at the early stage of AD [26]. The upregulation of MMP-9 could cause anti-Aβ immunotherapy to result in brain hemorrhages, which in turn contribute to the breakdown of the blood-brain barrier (BBB) [27,28,29], while the downregulation of activity and/or expression of MMP-9 could facilitate Aβ elimination across the BBB [30]. In addition, since upregulated of MMP-2 has been observed along with neurofibrillary tangles in neurons, it has been suggested that MMP-2 could stimulate tau and aggregation of hyperphosphorylated tau [31]. Along with MMP-2 and MMP-9, recent research indicated a high level of MMP-13 in the brain of AD patients highlights its implication in AD. Moreover, downregulating the activity of MMP-13 in AD mice could ameliorate amyloid pathology and cognitive deficits [32,33].

### 1.2. Regulators of Matrix Metalloproteinases

Over the decades, several inhibitors of MMPs have been developed. Many synthetic chemical agents targeting the active site of MMPs have failed to reach clinical trials due to significant dose-limiting musculoskeletal toxicity and/or ineffectiveness [34]. Moreover, the newly invented bio-engineered tissue inhibitors of metalloproteinases (TIMPs) or antibodies need further in vivo study of biosafety and reproducibility [35].

In nature, the enzymatic activity of MMPs is tightly regulated by the endogenous TIMPs through negative feedback by inhibition of MMPs [36,37]. TIMP-2, TIMP-3, and TIMP-4 could regulate the action of MMP-2, while TIMP-1 and TIMP-3 could regulate that of MMP-9 [38,39]. Usually, TIMPs could inactivate MMPs by (i) chelating Zn(II) from the active site of MMPs and (ii) interacting with the nucleophilic Glu located at the catalytic cleft of MMPs [40,41]. As TIMPs are natural inhibitors of MMPs, using engineered N-terminal TIMPs would be a promising strategy to develop MMP inhibitors [41]. With the help of computational design, some N-terminal of TIMP-2 variants were screened. For example, **N-TIMP2_WT_**, **N-TIMP2_A_**, **N-TIMP2_B_**, **N-TIMP2_C_**, and **N-TIMP2_D_** exhibited a significant 40–80% inhibition of MMP-2 and MMP-9 activity. However, they have only been tested in model systems to provide vital clues about the effectiveness of the drugs [42].

**Figure 1:**
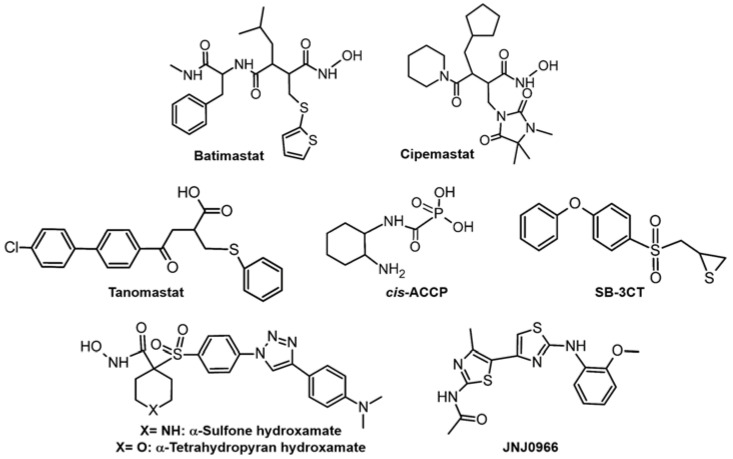
ZBG-based regulators and non-ZBG-based regulators targeting catalytic domain and exosite-targeted regulator.

To date, several strategies have been applied to develop regulators of MMPs. Those synthetic agents mainly focus on reducing the activity of MMPs by binding to (i) Zn(II) binding site, (ii) catalytic [non-Zn(II) binding] domain, and (iii) allosteric site and exosites. First, the regulators contain a Zn-binding group (ZBG), which can coordinate Zn(II) at the active site to interfere with the enzymatic activity [43]. ZBG-based regulators are the first generation of MMPs inhibitors, which are broad-spectrum inhibitors with poor selectivity and risk of side effects [44]. Some primary examples of MMP-2 and MMP-9 inhibitors based on ZBG have (i) hydroxamate (e.g., **Batimastat** has been shown to inhibit tumor growth and metastasis by regulating the activity of MMPs [45,46] and **Cipemastat**, a low molecule weight MMP inhibitor, has been shown to reduce tumor growth; Figure 1, top row) [47], (ii) carboxylate (e.g., **Tanomastat**, an analog of biphenyl non-peptide butanoic acid, has been indicated to against gelatinase A and B, and showed potential in phase I cancer treatment; however, it showed a very poor survival rate against small-cell lung cancer patients in clinical trials; Figure 1, middle row, left) [48], (iii) phosphinate moieties (e.g., ***cis*-ACCP**, an inhibitor of MMP-2/9, was found to act as an anti-metastasis drug; Figure 1, middle row, middle) [49], and (iv) thiolate (e.g., **SB-3CT**, an MMP-2/9 inhibitor, was shown to reduce tumor burden and improve anti-tumor immunity; Figure 1, middle row, right) [50]. Although these molecules have been developed to control the activity of MMPs by interfering Zn(II)-binding of MMPs, they need to reduce the musculoskeletal toxicity for further application [34].

The catalytic domain (non-Zn(II) binding), apart from Zn(II)-binding site, contains other recognition pockets, such as S1′ and S3′ pockets, which are highly selective between MMPs based on their size and depth. S1′ pocket of MMP-2 and MMP-9 are attracting inhibitors with a medium P1′ substituent [51]. For instance, a family of clicked hydroxamates such as α-sulfone and α-tetrahydropyran (Figure 1, bottom row, left) were found with potent inhibition of MMP-2 (with selectivity for MMP-2 over the closely related MMP-9) [52]. Nevertheless, since the flexibility and depth of the S1′ pocket have not been fully understood, there are no perfect MMP-2 or MMP-9 inhibitors developed based on this strategy [51].

Regulators targeting exosites and allosteric sites of MMPs have been also invented. Exosites and allosteric sites refer to the domains remote from the catalytic domain of MMPs, including the hemopexin domain, collagen domain, and pro-peptide domain. The inhibitors could bind to those sites and induce conformational changes or deformation of enzymes. For example, **JNJ0966** (Figure 1, bottom row, right) was proposed as a potent allosteric inhibitor of MMP-9 activation by binding to the structural pocket in proximity to the MMP-9 zymogen cleavage site [53]. Some structural-based inhibitory peptides for MMP-2 and MMP-9 were also introduced; 8-amino acid peptides (**IS4** and **IVS4** peptides that mimic the outer β-strand of Blade IV of the PEX domain of MMP-9) target hemopexin domain of MMP-9 and **P713** peptide targets collagen-binding domain of MMP-2 [54,55].

In addition, monoclonal antibodies have been applied as MMP inhibitors. For example, **GS-5745** is a highly selective antibody of MMP-9, targeting the residues (Arg162, Glu111, Asp113, and Ile198) near catalytic Zn(II) with no dose-limiting toxicity [56]. **GS-5745** has gone into clinical trials (NCT03631836, NCT02864381, NCT02520284, NCT02545504, and NCT01831427) for the treatment of gastric cancer (phase III), colitis, and Crohn’s disease (phase II), solid tumors, rheumatoid arthritis, and chronic obstructive pulmonary disease (phase I). Since the use of antibodies is restricted to intravenous or subcutaneous administration rather than oral administration, those antibody-based treatments may not be the perfect and first option for regulating MMPs in the human body. Therefore, further research should be performed to improve antibody usage and to evaluate biosafety and reproducibility in vivo [35].

Besides the mentioned inhibitors above, metal complexes have been applied to reduce side effects and improve the selectivity of molecules to MMPs. Along with the multiple metal complexes that have anti-tumor ability targeting DNA/RNA or mitochondria and anti-Aβ aggregation ability to reduce the risk of onset and/or progression of cancers and AD [57,58,59,60,61,62,63,64,65,66,67,68,69,70,71], various metal complexes (e.g., Pt-, Ru-, Fe-, Cu-, Au-, Ni-, Zn, Sn-complexes) could be applied as regulators for both expression and enzymatic activity of MMPs. Those metal complexes could be applied as cancer and AD treatments later. Therefore, in this review, we summarize the previously reported metal complexes as regulators for the activity and/or expression of MMP-2 and MMP-9.

## 2. MMP Regulators–Metal Complexes

**Figure 2:**
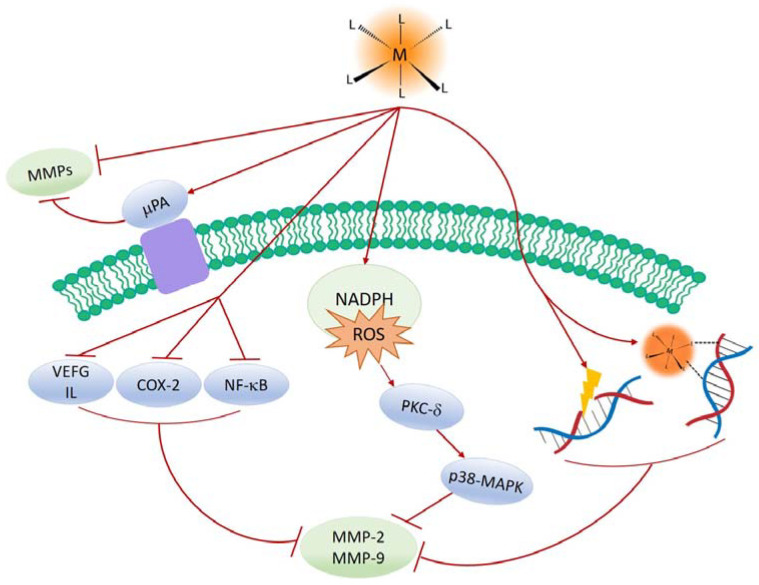
The proposed mechanisms for regulating MMPs by metal complexes in directly and indirectly.

Unlike the mentioned MMPs regulators that mainly inhibit the activity of MMPs, metal complexes could decrease in MMPs expression and activity to care for and cure cancers and AD [72,73,74,75,76]. The detailed mechanisms of MMPs regulation by metal complexes have not been fully revealed. Although the mechanisms are varied based on the types of metal ions and ligands, the proposed procedures for the inhibitory activity of metal complexes against MMPs are shown in Figure 2. The metal complexes summarized in this review could regulate the expression and activity of MMP-2 and MMP-9 through (i) direct binding to DNA and MMPs [77,78,79], (ii) generating ROS to damage DNA [80,81,82,83], (iii) activating PKC-δ via NADPH ROS generation to activate p38-MARK [84,85,86], (iv) inactivation of interleukins (IL) and vascular endothelial growth factor (VEGF) [87], and (v) suppression of COX-2 and NF-κB signaling [88,89,90].

### 2.1. Pt Complexes

Pt(II) complexes, including **cisplatin** and its derivatives, are widely used as anti-cancer agents. Since the first Pt(II) complex as anti-cancer agent, **cisplatin**, showed side effects and toxicity on normal cells, numerous Pt(II) complexes have been developed to have lower cytotoxicity and stronger anti-tumor activity than **cisplatin** [91,92,93,94,95]. Moreover, as MMPs are related to the onset and progression of cancers, regulation of MMPs activity and expression by Pt(II) complexes has been suggested as a potent strategy in cancer and AD treatment. 

#### 2.1.1. Pt(II) Complexes

**Figure 3:**
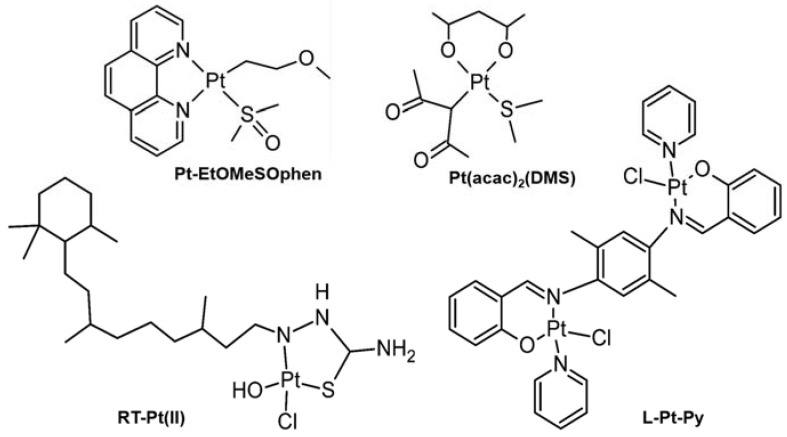
Pt(II) complexes regulating the activity and expression of MMPs.

A Pt(II) complex, **Pt-EtOMeSOphen** (Figure 3, top left), has been investigated for its anti-metastatic potential in various neuroblastoma cell lines [SH-SY5Y, SK-N-SH, and SK-N-BE(2)]. After a 24 h treatment of **Pt-EtOMeSOphen**, both MMP-2 and MMP-9 expressions and secretion were decreased dramatically in those cells [85]. **Pt(acac)_2_(DMS)** (Figure 3, top right) was revealed to reduce NHE1 activity, inhibit cell migration and invasion, and decrease expression and activity of MMPs in breast cancer cells (MCF-7) and SH-SY5Y. With a treatment of 0.5 μM of **Pt(acac)_2_(DMS)**, the expression and activity of MMP-2 and MMP-9 in SH-SY5Y cells were significantly decreased [86].

Retinoic acid-Pt(II) Complex, **RT-Pt(II)** (Figure 3, bottom left), was investigated as an anti-inflammatory agent, which could regulate the expression of NF-κB signaling pathway and the level of IL-1β, IL-6, IL-8, MMP-1, and MMP-13 in synovial fluid of mice [96]. **RT-Pt(II)** treatment (4 ng/mL) to Rheumatoid synovial cells (MH7A), the expression of NF-κB mRNA was decreased dramatically. Furthermore, the expression of MMP-9 could be regulated via NF-κB inhibition [97]. Moreover, with the treatment of **RT-Pt(II)** on rats for 21 days (dosed of 2–5 mg/kg), both NF-κB and active MMP-13 levels were decreased. Besides, it has been proposed that downregulating MMP-13 activity through pharmacological inhibition could have therapeutic potential for treating AD [33].

Pt(II) dimetallic square planner complex containing mixed ligands (**L-Pt-Py**; L=*p*-phenylenediamine (2-hydroxyltoluene)_2_; Figure 3, bottom right), confirmed for its ability to suppress MMPs expression and inhibits the growth, invasion, and migration of cancer cells [98]. **L-Pt-Py** was examined in human lung cancer cells (A549) and MCF-7 at a dose of 10 μM for 24 h, showing a strong decrease in MMP-2 and MMP-9 RNA expression [98].

#### 2.1.2. Pt(IV) Complexes

**Figure 4:**
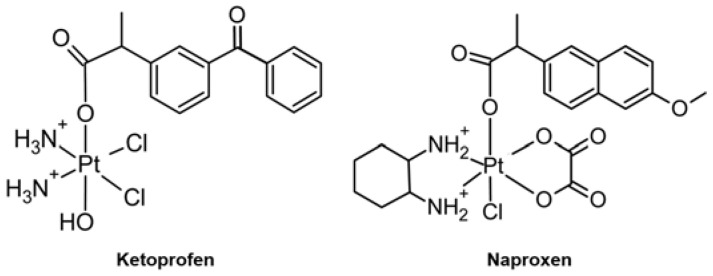
Pt(IV) complexes regulating the activity and expression of MMPs.

Compared to Pt(II) complexes, Pt(IV) complexes with octahedral geometry could undergo fewer off-target reactions and side effects with less toxicity [93,99]. A new series of Pt(IV) complexes, such as **Ketoprofen** (Figure 4, left), was designed for chemotherapeutic and immunotherapeutic effects targeting MMPs, cyclooxygenases (COXs), and programmed death ligand 1 (PD-L1). The expression of MMP-9 in A549 cells was significantly reduced after a 24 h treatment with 10 μM of **Ketoprofen** [88]. **Naproxen** (Figure 4, right) was designed as a potent inhibitor of both MMP-9 and COXs. The MMP-9 expression in tumor tissues was downregulated with an inhibitory greater than that of **oxaliplatin**, which could be ascribed to the incorporation of the **naproxen** ligand [89].

### 2.2. Ru Complexes

Ru complexes have been proposed as potent anti-cancer agents as well [100]. Compared to Pt complexes, Ru complexes presented multiple advantages; (i) transported by Fe transporters such as transferrin, (ii) high selectivity due to its octahedral geometry, (iii) easy transformation of oxidation states, +2, +3, and +4, and (iv) more favorable ligand exchange kinetics. Moreover, Ru(II) complexes are believed to inhibit the expression and activity of MMP-2 and MMP-9 with low toxicity [101,102,103,104]. Ru ions can be found in two stable oxidation states in physiological conditions, Ru(II) and Ru(III), both of the oxidation states to support a six-coordinate octahedral structure. Nevertheless, they could be coordinated to different geometries by ligands, then could contribute to various biological redox reactions [105,106].

#### 2.2.1. Ru(II) Complexes

**Figure 5:**
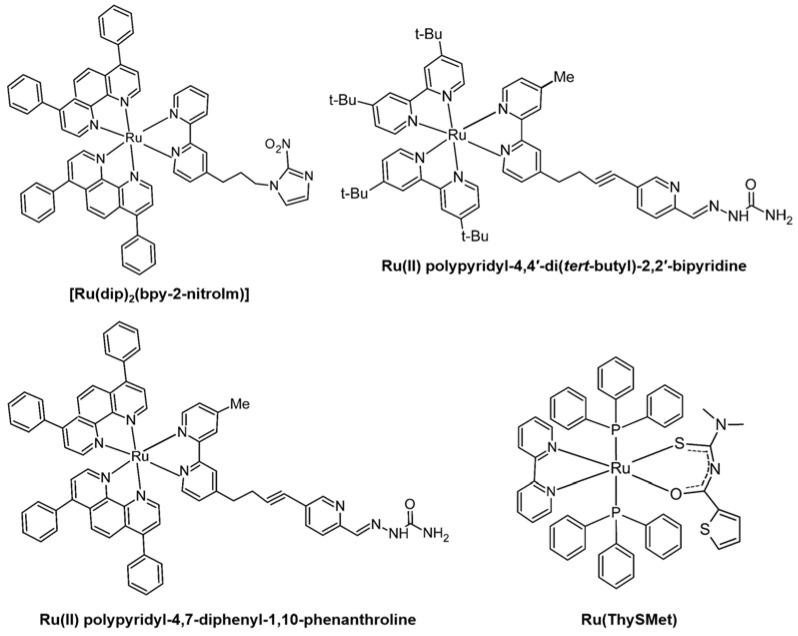
Ru(II) complexes regulating the activity and expression of MMPs.

[Ru(dip)_2_(bpy-2-nitroIm)] (Im=imidazole; Figure 5, top left) has been proposed to be an effective anti-cancer agent against breast cancer cells (4T-1) and A549. The complex could significantly decrease MMP-9 gene expression in 4T-1 cells after a 24 h treatment under both normoxic and hypoxic conditions [107]. Meanwhile, [Ru(dip)_2_(bpy)]^2+^ was indicated to strongly increase MMP-9 mRNA expression in 4T-1 cells after a 24 h treatment under normoxic conditions, but a strong decrease in hypoxic condition [107].

Two Ru(II) polypyridyl complexes containing 2,2′-bipyridine substituted by a semicarbazone 2-formylopyridine moiety and 4,4′-di(*tert*-butyl)-2,2′-dpyridyl (Figure 5, top right) or 4,7-diphenyl-1,10-phenanthroline (Figure 5, bottom left) as auxiliary ligands were proved to against the development of metastasis via inhibiting MMPs activity. The cell-associated MMPs activity was investigated by measuring the proteolysis of FS-6. As **GM6001** (a broad-spectrum hydroxamate metalloproteinase inhibitor) does, the Ru(II) polypyridyl complexes strongly inhibit the activity of MMPs in A549 cells. Moreover, those complexes presented ca. 20–85 times higher inhibition of MMP-2 over **GM6001,** while the effectiveness in inhibition of MMP-9 was lower than **GM6001** [108]. **Ru(ThySMet)** (Figure 5, bottom right) was revealed to significantly inhibit the activity of MMP-9 in T4-1 cells by eight folds compared with untreated cells and was 4-folds lower than **PD98059** (a MEK inhibitor, which was used as positive control) treatment [90]. **Ru(ThySMet)** could inhibit the activity of MMP-9, possibly by reducing the transcription of MMP-9 by decreasing the NF-κB expression [109].

#### 2.2.2. Ru(III) Complexes

Under physiological conditions, Ru(III) complexes can be reduced to Ru(II) complexes and still retain their octahedral ligand sets [110]. The anti-cancer activity of Ru(III) complexes depends on the reduction of Ru(III) to Ru(II) [111]. Ru(III) complexes could also mimic Fe metabolisms, enabling them to overcome the high selectivity of transferrin receptors on cancer cells [112]. Thus, various Ru(III) complexes have entered clinical trials in different phases of cancer treatment.

**Figure 6:**
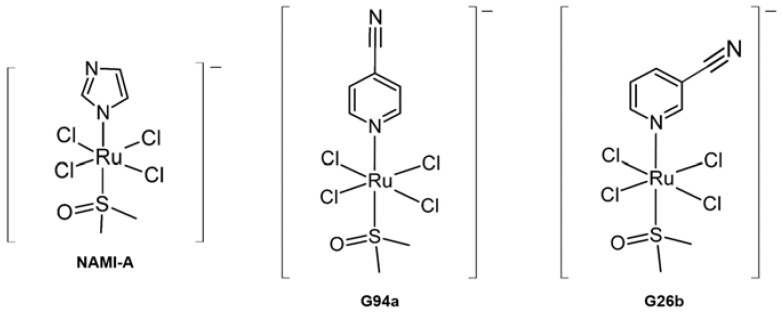
Ru(III) complexes regulating the activity and expression of MMPs.

**NAMI-A** (Figure 6, left) has been revealed to greatly reduce lung metastasis and the formation of solid metastasizing tumors in mice [113]. **NAMI-A** has a pseudo-octahedral Ru(III) center surrounded by four chloride ligands in the equatorial plane [114]. There were the following multiple perspectives about the anti-cancer mechanism of **NAMI-A** and its derivatives: (i) interacting with DNA forming the bifunctional intrastrand adducts on double helical DNA [115], (ii) mimicking Fe(III) behaviors to overcome the selective transferrin recognition to the entry of cancer cells [116], (iii) acting as anti-angiogenic agent [117], (iv) binding to collagen of the extracellular matrix and cell surface integrins [112]. Moreover, **NAMI-A** has been proposed to reduce invadopodia in tumor cells where MMP-9 is preferentially localized in active form and decrease the secretion of MMP-9 [77]. Recently, **NAMI-A** derivatives containing pyridine, i.e., **G94a** and **G26b** (Figure 6), could regulate the transcription of MMP-2 and MMP-9 in MCF-7 cells efficiently. **G94a** reduced the expression of MMP-2 by 80% and MMP-9 by 70%, whereas **G26b** reduced the expression of MMP-2 and MMP-9 genes by 80%. Particularly, **G26b** was shown to be more effective in inhibiting the transcription of MMP-2 over **NAMI-A** [78].

Furthermore, **NAMI-A** and its derivatives containing pyridine (**Ru-N**) were also proposed to undergo ligand exchange and covalent binding to the Aβ, leading to the modulation of Aβ aggregation and the formation of high molecular weight aggregates, amorphous, and fibrillary aggregates [118]. Therefore, Ru(III) complexes could be applied to treat AD as well.

### 2.3. Au Complexes

**Figure 7:**
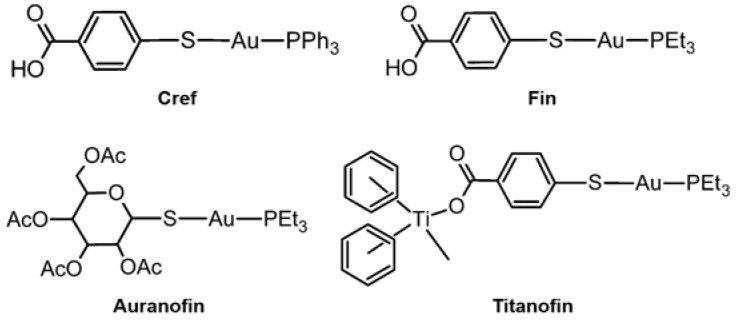
Au complexes for regulating the activity and expression of MMPs.

**Cref** (Figure 7, top left), an Au monometallic complex, was indicated to act as a regulator of MMP-2 expression. **Cref** decreases the MMP-2 expression on human renal cancer cells (Caki-1) by about 50% after a 72 h treatment. **Cref** also showed a small effect in downregulating the expression of MMP-9 in Caki-1 cells [87]. **Fin** (Figure 7, top right) also could downregulate the expression of MMP-2 on Caki-1 cells but had no influence on MMP-9 expression. **Auranofin** (Figure 7, bottom left) was revealed for its anti-metastatic ability by downregulating the expression of both MMP-2 and MMP-9 on Caki-1 cells after a 72 h treatment [87]. Titanocene–Au complexes [(η-C_5_H_5_)_2_TiMe(μ-mba)Au(PR_3_)] (PR_3_=PPh_3_: **Titanocref** and PR_3_=Pet_3_: **Titanofin**; Figure 7, bottom right) were recently investigated as well. **Titanocref** and **Titanofin** effectively inhibited the expression of MMP-9 in Caki-1 cells by 90% after a 72 h treatment. Those complexes, however, had no effective inhibiting the expression of MMP-2 [87].

### 2.4. Fe Complexes

**Figure 8:**
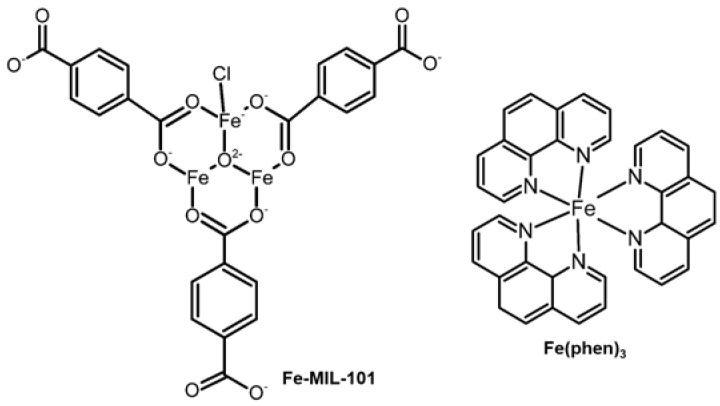
Fe complexes regulating the activity and expression of MMPs.

**Fe-MIL-101** (Figure 8, left), was developed against the proliferative effect on HeLa, A549, human ovarian cancer cells (SK-OV-3), and human umbilical vein endothelial cells (HUVEC). It could restrict ovarian tumor growth without significant toxicity [119]. A lower amount of both MMP-2 and MMP-9 was observed in SK-OV-3 and HUVEC cells after a 24 h treatment of **Fe-MIL-101**. Moreover, **Fe-MIL-101** significantly inhibited the vascular endothelial growth factor (VEGF) or conditioned media (CM), which is involved in the upregulation of MMP-2 and MMP-9 expression and activity. Unfortunately, the MMP regulation mechanism of **Fe-MIL-101** remains unclear [119]. [**Fe(phen)_3_**]^2+^ (Figure 8, right), a Fe(II) polypyridyl complex with planarity geometry of the ligands in complex, was proposed to be an anti-metastatic agent via regulating MMP-2 and MMP-9. A significant decrease in both MMP-2 and MMP-9 expression with an increase in TIMP-1 was observed in human glioma cells (U87) after a 24 h treatment [84].

### 2.5. Cu Complexes

Multiple Cu complexes have been evaluated for therapeutic indications, including cancers and AD as well. Cu complexes have been recognized as a limiting factor for the progression of cancer, including growth, angiogenesis, and metastasis via regulation of MMPs activity and expression level [120].

**Figure 9:**
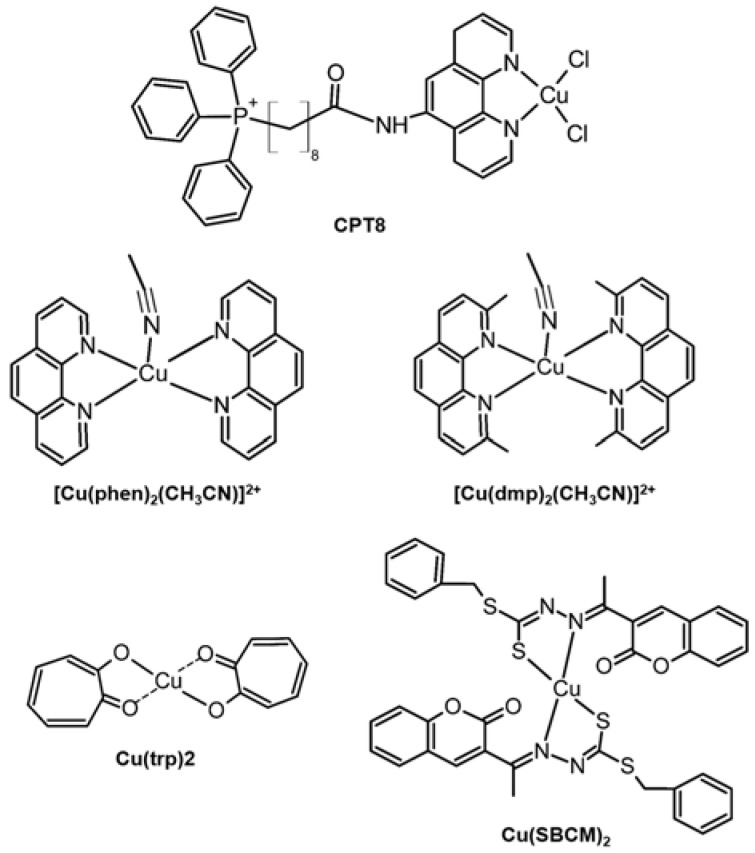
Cu complexes regulating the activity and expression of MMPs.

Phenanthroline containing Cu(II) complex, **CPT8** (Figure 9, top), could downregulate the expression of MMP-2 in SK-OV-3 cells [121]. [Cu(phen)_2_(CH_3_CN)]^2+^ and [Cu(dmp)_2_(CH_3_CN)]^2+^ (Figure 9, middle row), which have a similar structure to [Cu(phen)_2_]^+^, were examined for its anti-cancer activities in 2D and 3D models of human osteosarcoma (MG-63), A549, MCF-7, triple-negative breast adenocarcinoma cells (MDA-MB-231), and colorectal cancer cells (HT-29, LS174T, and Caco-2) [80,122]. Similar to [Cu(phen)_2_]^+^, the anti-cancer activity of [Cu(phen)_2_(CH_3_CN)]^2+^ and [Cu(dmp)_2_(CH_3_CN)]^2+^ was attributed to the ROS production by nucleolytic activity of those Cu(II) complexes [80,122]. Otherwise, the anti-cancer activity of the two Cu(II) complexes was also contributed by the inhibition of the NF-κB pathway and MMPs expression. [Cu(phen)_2_(CH_3_CN)]^2+^ could decrease MMP-2 and MMP-9 expression in HT-29 cells significantly [81]. [Cu(dmp)_2_(CH_3_CN)]^2+^, containing methyl substituents in the *phen* ligand, has a stronger anti-cancer activity than [Cu(phen)_2_(CH_3_CN)]^2+^. [Cu(dmp)_2_(CH_3_CN)]^2+^ inactivated the NF-κB pathway in both monolayer and spheroids, leading to the downregulation of MMP-2 and MMP-9 production in HT-29 cells by 75% and 50%, respectively [80].

[Cu(trp)_2_] (Figure 9, bottom left) was demonstrated to inhibit metastatic progression in human breast cancer cell lines, MCF-17 and MDA-MB-231, by regulating MMP-2 and MMP-9 expression. The secretion level of MMP-2 and MMP-9 in MDA-MB-231 cells was significantly reduced after incubation with 4 μM of [Cu(trp)_2_] for 48 h [82]. [Cu(SBCM)_2_], synthesized with S-benzyldithiocarbazate and 3-acetylcoumarin (Figure 9, bottom right), indicated its safety at low concentrations. Besides, [Cu(SBCM)_2_] could significantly downregulate the expression of MMP-2 in MDA-MB-231 cells [123].

### 2.6. Other Metal Complexes

**Figure 10:**
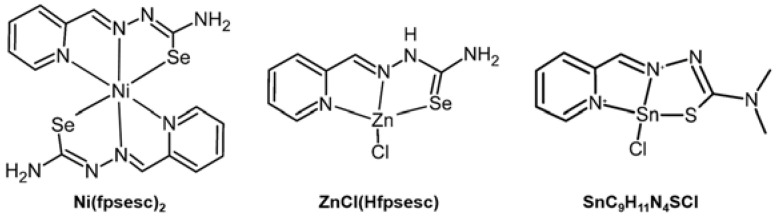
Ni(II)-, Zn(II)-, and Sn(II)-complexes regulating the activity and expression of MMPs.

Ni(II) complex composed of 2-formylpyridine selenosemicarbazone, **Ni(fpsesc)_2_** (Figure 10, left), showed different effects on MMPs activity in HeLa, MDA-MB-361, EA.hy 926 cells, and mouse endothelial MS cells. After a 6 h treatment of **Ni(fpsesc)_2_**, the activity of MMP-2 was significantly decreased in all three cell lines, while the activity of MMP-9 was upregulated in HeLa and MS cells and downregulated in EA.hy926 cells [83].

Zn(II) complex containing 2-formylpyridine selenosemicarbazone, **ZnCl(Hfpsesc)** (Figure 10, middle), could lower the level of the active form of MMP-2 in HeLa, MDA-MB-361, and mouse endothelial MS cells, while increasing the MMP-2 activity in human endothelial EA.hy926 cells. Moreover, the activity of MMP-9 was increased significantly in HeLa cells upon treatment of the **ZnCl(Hfpsesc)** [83].

An Sn(II) complex, [SnC_9_H_11_N_4_SCl] (Figure 10, right), demonstrated its inhibiting ability against MMP-2 activity potentially by (i) binding of Sn(II) complex to MMP-2 by forming a hydrogen bond with Leu83 in the S1 pocket or (ii) replacing Zn(II) in the P1 pocket to form complexation with His120 or His130 and forms a hydrogen bond with His85 [79].

## 3. Conclusions

As we summarized above, many different metal complexes have been developed, and examined their potent application in treating fatal human diseases, cancers, and AD. Since MMPs are involved in the pathology of those diseases, various molecules have been applied to regulate the expression and activity of MMPs. Particularly, metal complexes targeting MMP-2 and MMP-9 have been reported as effective regulators. As shown in Table 2, the advantages and limitations of metal complexes as regulators of MMPs are summarized. Through this review, we provided the current studies and knowledge of the metal complexes targeting MMPs and their potentials for treating fatal human diseases. This approach could be an important aspect for the care of and treatment cancers and AD by regulating MMPs. Further, less toxic and more effective metal complexes for regulating the expression levels and activity of MMPs should be developed for successful treatments of fatal diseases in the future.

## Figures and Tables

**Table 1:** Locations and biological functions of MMP-2 and MMP-9 as well as the diseases related to the enzymes.

MMP	Expressed Cells	Biological Functions	Related Diseases
**MMP-2**	Cardiomyocyte Smooth muscle Platelet Fibroblast Osteosarcoma Cerebral neuron Endothelial cell Keratinocyte	Anti-inflammation Cell migration through activate p38-MAPK/ML/SHP27 signaling Elevation of collagen affinity, epithelial cell migration Enhancement of transforming growth factor-β (TGF-β) Neuronal apoptosis and growth of axons Proliferation and cleaving of IGF-1 binding proteins Facilitation of Aβ clearance	Cancer Alzheimer’s disease Inflammation Asthma Fibrosis Cardiovascular disease
**MMP-9**	Leukocyte Cardiomyocyte Retinal endothelial cell Osteoclast Cerebral neuron Megakaryocyte	Anti-inflammatory and pro-inflammatory activity Increase in collagen affinity Reduction of IL-2 response Activation of TGF-β in cancer progression Apoptosis of hypertrophic chondrocytes Increase in sAPPα and facilitation of Aβ clearance	Cancer Alzheimer’s disease Inflammation

**Table 2:** A summary of general advantages and limitations of metal complexes mentioned above.

Anti-Cancer and Alzheimer’s Disease Related Actions	Advantages	Limitations
Regulation of the expression and activity of MMP-2 and MMP-9 Anti-metastasis Anti-angiogenesis Anti-proliferative Suppression of cell migration Promotion of apoptotic process	Simplicity in synthesis Effective at a small dose, in a short time Higher cytotoxicity towards cancer cells with less side effects than **cisplatin** Stronger MMPs inhibition than the hydroxamate-based MMPs inhibitors (**GM6001**) Higher selectivity against towards cancer cells with less toxicity to normal cells (e.g., **Ru(ThySMet)** [90]; Fe complexes [84,119]; **CPT8** [121]; [Cu(trp)_2_]^2+^ [82]) Under clinical trial (e.g., **NAMI-A**). No significant damage or toxicity to the major organs (e.g., Ru complex [77,78]; Fe complexes [84,119])	Unclear detailed mechanisms of cytotoxicity Some side effects (e.g., Ru(dip)_2_(bpy-2-nitroIm) [107])

## Data Availability

Not applicable.
